# Everybody's Page

**Published:** 1891-04-18

**Authors:** 


### EVERYBODY'S PAGE.

It is said that more than 5 per cent. of the total strength of the army is constantly ineffective from sickness.

Rumour says that "General" Booth is going to name the matches made at his Hackney match manufactory "Salvation Blazes."

There is a story told of Thackeray that he was found one morning in Paris, putting some Napoleons into a pill box, on the lid of which was written "one to be taken occasionally." "What are you doing?" he was asked, "Well," he replied, "there is an old person here who is very ill and in deep distress, and I expect this is the kind of medicine she wants. Dr. Thackeray intends to leave it with her himself."

It was formerly compulsory that all persons should be buried in flannel gowns, and there was an Act of Parliament to that effect. The nearest relatives of the deceased person had to go before a magistrate and make oath that the body was buried according to the Act of Parliament, that is, in woollen. The object of this decree was the benefit of the wool trade, and our ancestors seem to have thought far more of the trade of the country than the cause of death of its inhabitants, for they possessed no registration.

The North Metropolitan Tramways Company have just begun, at the suggestion of Mr. Marcus Adler, a cheap service at stated times throughout the day, affording special accommodation to workpeople carrying parcels. This concession will undoubtedly prove a great boon to dwellers in the East-end, where omnibus fares are abnormally high, being on the average more than double those in the West-end. It will probably also be a benefit to the shareholders, who will find that the additional traffic which they thereby secure will not curtail their profits. But this is not the reason which urged Mr. Adler to press for this concession. He looks to the universal introduction of cheap fares as the saviour of congested towns, but surely he ascribes a too magical power to them. Even if they attract working men from their unhealthy homes in the congested districts in our large towns into the country, the evil cannot be cured unless the laws against overcrowding are most strictly enforced and healthy and attractive homes are provided in the country. The tramways do not help much to induce people to leave towns, and we must look to the example set by the Great Eastern Railway being followed by the other railway companies to help to solve the pressing problem.

Dr. Edgar Sheppard, medical authority of Colney Hatch, says that thirty-five to forty out of every hundred are in lunatic asylums through drink.

That devastator the moth, whose ravages are so dreaded, may be stopped on his mad career among woollens generally by plentifully sprinkling ordinary dry common salt among them before putting them by.

"My frens, de aiverage man comes mighty nigh being an idiot in takin' car' of himself. You have seen him wearin' a fur cap on his head, while his shoes let in de snow and water. He wears an obercoat on his back and nuffin but a thin shirt over his chist. He's mighty skeert about freezin' his fingers, while his throat is exposed to blizzards. An so he's ailin'! ailin'!!" We commend this short extract to the consideration of our readers, whilst these north-east winds are about.

It is stated on good authority that the annual consumption of coffee in the world amounts to 856,000 tons. The United Kingdom compares unfavourably with other countries as only 14,000 tons are used in it. Coffee grows in favour in this country among all classes, but it would be in far more general use were it properly made. That terrible beverage so often handed to us as coffee consisting principally of chicory and hot water, is a trial to any digestion, but coffee roasted and ground when required (not prepared a month before it is wanted) is one of the most refreshing drinks.

The Public Health Bill ought to bring us a step nearer good sanitation. It will increase the number of sanitary inspectors, it forbids the emptying of dust-bins during the day and increases the supervision both over the food and the dwellings of the poor. So far so good, but how long will it be before these various enactments become practically dead letters? It is not so much new laws that are wanted to increase our sanitary well-being, but the carrying out of old ones. The emptying of dust-bins by night is a distinct improvement, but perhaps the day will soon come when the dust-bin itself will vanish and refuse will be carted away every day. Can there be a greater danger conceived than the dust-bins in some of these great blocks of flats which are rapidly spreading over London, a huge collection of decomposing matter carted away once, or perhaps twice a-week? Sanitarians while they allow this will strain at a gnat and swallow a camel.

